# Pyrethroid Resistance Reduces the Efficacy of Space Sprays for Dengue Control on the Island of Martinique (Caribbean)

**DOI:** 10.1371/journal.pntd.0001202

**Published:** 2011-06-21

**Authors:** Sébastien Marcombe, Frédéric Darriet, Michel Tolosa, Philip Agnew, Stéphane Duchon, Manuel Etienne, Marie Michèle Yp Tcha, Fabrice Chandre, Vincent Corbel, André Yébakima

**Affiliations:** 1 Institut de Recherche pour le Développement (IRD), UR016 CCPV/UMR MIVEGEC, Montpellier, France; 2 Entente Interdépartementale pour la Démoustication du Littoral Méditerranéen, Montpellier, France; 3 Centre National de la Recherche Scientifique, GEMI, Montpellier, France; 4 Conseil Général de la Martinique, Fort-de-France, France; 5 Centre de Recherche Entomologique de Cotonou (CREC), Cotonou, Bénin; Mahidol University, Thailand

## Abstract

**Background:**

Dengue fever is reemerging on the island of Martinique and is a serious threat for the human population. During dengue epidemics, adult *Aedes aegypti* control with pyrethroid space sprays is implemented in order to rapidly reduce transmission. Unfortunately, vector control programs are facing operational challenges with the emergence of pyrethroid resistant *Ae. aegypti* populations.

**Methodology/Principal Findings:**

To assess the impact of pyrethroid resistance on the efficacy of treatments, applications of deltamethrin and natural pyrethrins were performed with vehicle-mounted thermal foggers in 9 localities of Martinique, where *Ae. aegypti* populations are strongly resistant to pyrethroids. Efficacy was assessed by monitoring mortality rates of naturally resistant and laboratory susceptible mosquitoes placed in sentinel cages. Before, during and after spraying, larval and adult densities were estimated. Results showed high mortality rates of susceptible sentinel mosquitoes treated with deltamethrin while resistant mosquitoes exhibited very low mortality. There was no reduction of either larval or adult *Ae. aegypti* population densities after treatments.

**Conclusions/Significance:**

This is the first documented evidence that pyrethroid resistance impedes dengue vector control using pyrethroid-based treatments. These results emphasize the need for alternative tools and strategies for dengue control programs.

## Author Summary

The mosquito *Aedes aegypti* is the major vector of the Dengue virus in human populations and is responsible of serious outbreaks worldwide. In most countries, vector control is implemented by the use of insecticides to reduce mosquito populations. During epidemics, insecticides of the pyrethroid family (blocking the voltage gated sodium channel protein in the nerve sheath) are used by space spraying with vehicle mounted thermal foggers to kill adult mosquitoes. Unfortunately some populations of *Ae. aegypti* have become resistant to these insecticides, leading to operational challenges for public health services. In Martinique (French West Indies), resistance to pyrethroids was detected in the 1990s. The present study assessed the impact of this resistance on the efficacy of vector control operations in 9 localities of Martinique. Here we showed that the resistance strongly reduces the efficacy of pyrethroid-based treatments, thus emphasizing the urgent need for alternative insecticides or tools to reduce dengue transmission.

## Introduction

Control of the vector *Aedes aegypti* remains the primary approach to reducing transmission of dengue and dengue hemorrhagic fever in human populations [1], [2]. Adult control is generally implemented by spraying chemicals into the air to reduce adult populations after larval control has failed to limit their density or virus transmission has reached outbreak levels [3]. However there has been considerable debate as to the efficacy of space spray applications for the control of dengue epidemics in the tropics [4]. Several studies found space spraying effectiveness as variable and often poor due to limited penetration of insecticides into dwellings [5], [6]. In contrast, Gratz [7] reported successful control of adult *Ae. aegypti* populations in south-east Asia where spatial applications were applied in a well organized manner (e.g. using adapted materials and formulations, effective application procedures and planning). Furthermore dengue control programs rely on just two of the four major classes of insecticides available for use in public health; pyrethroids and organophosphates. The spread of resistance to these two chemical classes is a major concern for dengue control in the tropics [8].

In Martinique (French Caribbean) 5 major dengue outbreaks have occurred in the last 15 years [9], and each only involved the vector *Ae. aegypti*. DDT and several organophosphates (e.g. malathion, fenitrothion) were used to control adults for decades, but in the early 1990s there was a switch to pyrethroids (i.e. deltamethrin and permethrin) because of their strong insecticidal properties at low application rates and for their safety in the environment and towards humans [10], [11]. Since then local populations of *Ae. aegypti* have developed high levels of resistance to pyrethroids [12]. This is due to high frequencies (>80%) of the “knock-down resistance” (kdr) mutation (V1016I), and elevated activity of detoxification enzymes (i.e. cytochrome P450 mono-oxygenases, glutathione-S-transferases and, carboxylesterases) [12]. Detox chip microarray and RT-qPCR validation showed an over-transcription of multiple detoxification genes, essentially P450s, in pyrethroid-resistant adults compared to their susceptible counterparts [12], [13]. This multiple resistance had a strong negative effect on the efficacy of pyrethroid and pyrethrins space sprays against natural *Ae. aegypti* mosquitoes in semi-field conditions (based on data from mosquito sentinel cages) [14]. This finding is all the more worrying as these two adulticides (deltamethrin and natural pyrethrins) remain the only insecticides available for the control of adult mosquitoes in Martinique due to European legislation regarding pesticide use and its application [15].

We conducted a medium-scale field trial in Martinique to assess the operational impact of pyrethroid-resistance on the efficacy of deltamethrin (1 g/Ha) and pyrethrins (10 g/Ha) ULV space sprays following WHO protocols [16], [17]. Our results demonstrate that the efficacy of pyrethroid and pyrethrins space sprays was strongly reduced when applied against natural populations of *Ae. aegypti* resistant to pyrethroids. This emphasizes the urgent need to develop alternative tools and strategies for the control of dengue vectors.

## Methods

### Study areas

**Figure 1:**
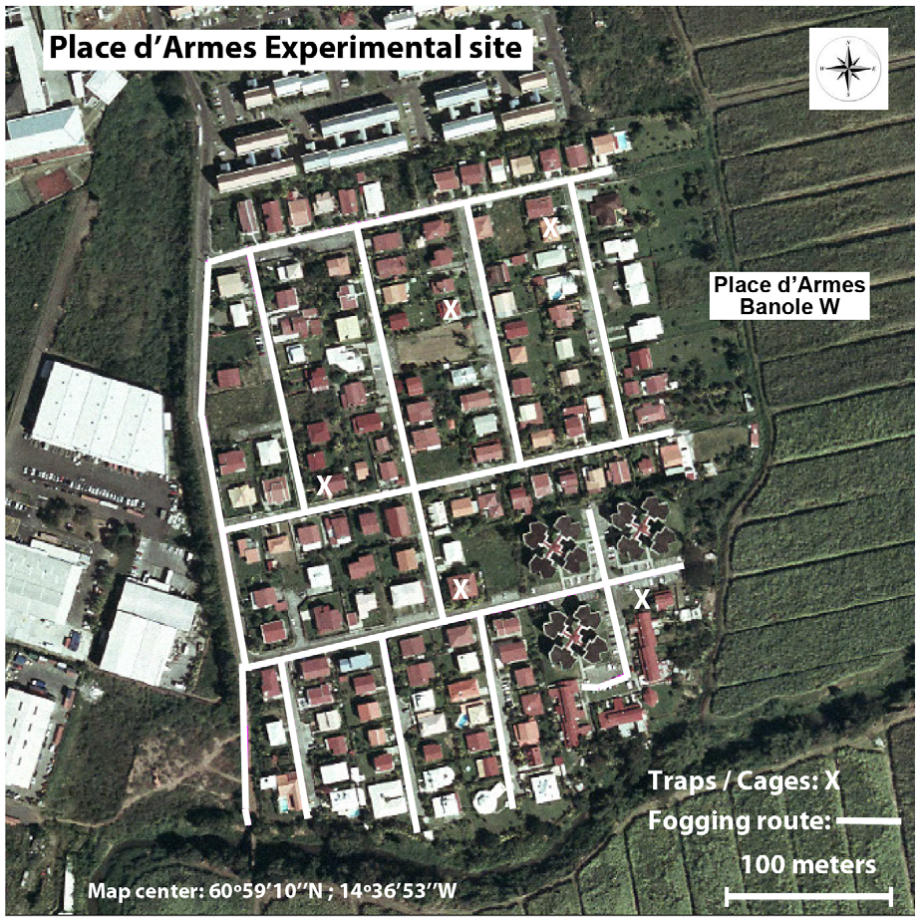
Route of space spraying and individual houses where traps and cages were placed. The efficacy of synergized pyrethrins and deltamethrin against *Aedes aegypti* was evaluated in field conditions by conducting space spray applications using 4x4 vehicles mounted with a thermal fogger in 9 localities of Martinique. In each site, 3 rounds of treatment were made at 2 day intervals. In each locality, 5 sentinel cages containing female mosquitoes were placed inside and outside the houses (white X). Electric mosquito traps were also placed on the inside and outside of the same houses. The locality shown is “Place d'Armes”, which was treated with BanoleW (Control).

The two districts of Lamentin and Ducos located in the western part of Martinique were selected for the field trial. Both have a dry tropical climate with a rainy season occurring between May and November and an annual precipitation of 2,500 mm. Five localities were selected in the districts of Ducos (14°34′0″N, 60°58′60″W): Bac, Durivage, Bois Neuf, Canal and Morne Carette. Four localities were chosen in Lamentin (14°36′0″N, 60°0′0″W): Grand Case, La Favorite, Place d'Armes and Long Pré. The localities were separated from each other by 1 to 3 km, thus minimizing interference between or among localities. The 9 localities were housing estates and hamlets, more or less urbanized, composed of ∼60–150 houses (Figure 1). The island of Martinique is an overseas department of France and subject to European law, including laws related to the use, application and handling of insecticides.

### Resistance status

One month before the field evaluation, larvae from the 9 localities were sampled and reared in the laboratory. Adult females of the F1 progeny were used in tarsal contact tests with treated filter paper and compared with the susceptible Bora strain. Tests were run using filter papers treated with technical grade deltamethrin (100% [w/w]; AgrEVO, Herts, United Kingdom) and pyrethrum (mixture of 6 pyrethrins [pyrethrin I, pyrethrin II, cinerin I, cinerin II, jasmolin I, jasmolin II]; 25.44% [w/w]; Pyrethrum Board of Kenya, Nakuru, Kenya) following WHO guidelines [18]. The resistance status of *Ae. aegypti* in each locality was determined by using discriminating dosages of deltamethrin (0.05%) and pyrethrum (1%) [19]. For each strain, five batches of 20 non-blood fed females (2–5 days old; n = 100) were exposed to the insecticides for 60 minutes and mortality recorded 24 hours later.

### ULV thermal fogging applications

The efficacy of synergized pyrethrins and deltamethrin was evaluated in field conditions by conducting space spray applications using three 4x4 vehicles mounted with a Curtis Dinafog MaxiPro4 thermal fogger. In each site, 3 rounds of treatment were made at 2 day intervals according to the treatment procedure used by the mosquito control unit of Martinique. Formulations of deltamethrin (K-Othrine ULV 15/5, 15% [w/v] +0.5% esbiothrine [w/v]; Bayer Environmental Science, Lyon, France) and synergized pyrethrins (AquaPy, 3% EW [w/v] +13.5% piperonyl butoxide (PBO) [w/v]) were used. These two insecticides are routinely used in Martinique and their use is authorized by European legislation [15]. The formulation of pyrethrins was mixed with water as a carrier for the thermal fogging, as recommended by the manufacturer, and deltamethrin was mixed with mineral oil (Banole W), which was also used alone as a control treatment. K-Othrine ULV 15/5 is the reference formulation that has been used for many years in Martinique for the control of *Ae. aegypti*. Each insecticide and their formulations were notified in the European Directive 98/8EC of 16 February 1998 [15].

The choice of localities was based on *Ae. aegypti* density (larval indices and electric traps) and resistance status. Three groups of 3 localities having similar entomological indices were formed and one locality of each group was allocated to an insecticide or control treatment; these groups acted as a blocking factor during data analysis. Following this procedure, the localities of Canal, Morne Carette and Grand Case were treated with deltamethrin; Bac, Bois Neuf and La Favorite were treated with pyrethrins; and Durivage, Long Pré and Place d'Armes were control sites and sprayed with Banole W. A single vehicle was allocated to each insecticide throughout the study.

Treatments were carried out in each locality at the period corresponding to the peak of mosquito flight activity (between 5:00 PM and 8:00 PM). Before treatment, the spraying apparatus was calibrated (i.e. flow rates were 450 mL water/min and 380 mL oil/min) and blank trials were made in each locality in order to estimate the length of routes and the quantity of formulation needed (Figure 1). During application, the speed of vehicles was between 8 and 10 km/hr, and the volume of mixture applied between 760 and 980 mL/Ha. With an efficient pulverization swath of 40 meters, the 9 areas treated were between 5.6 and 10.6 Ha. During application a hand-held anemometer (TFA) recorded temperature, wind speed and its direction. Temperatures and relative humidity were recorded constantly over the period of experiment at each locality using a Hobo Pro v2 probe. Precipitation was recorded daily with a meteorological unit (Auria 4) placed in Lamentin and provided by the General Council of Martinique. Operators and people involved in the evaluation were informed as how to handle insecticides and the corresponding safety procedures. Operators used protective clothing, shoes and facemasks to reduce the risk of exposure to insecticides. The sprays performed in our study were part of routine spraying campaigns in Martinique organized by French governmental authorities. The spraying procedures used in this study (i.e. trucks, foggers, asking people to open their doors and windows, etc.) were the same as those used by French governmental authorities in dengue control programs in Martinique. The experiments were validated by the health departments, the mayors of the two municipalities (Ducos and Lamentin), and by the French Agency for Environmental Health and Safety (AFSSET). Before the experiments, inhabitants of each locality received a brochure with information explaining the treatments and when they would be applied. People living in individual residences where cages and traps were installed were asked for their permission to do so and the details of each location were recorded by the vector control staff of Martinique. All the inhabitants gave their oral consent prior to treatments; their names, addresses and phone numbers were recorded by the vector control staff and are held at the offices of the Conseil Général de la Martinique, Fort-de-France, France.

### Caged female mortality

Insecticidal activity of pyrethroid space sprays was evaluated against a susceptible reference strain (Bora) on 3 separate occasions according to the WHO cage bioassay method [16]. Cylindrical steel frame cages (90 mm diameter x 153 mm height) covered on all three walls with a mosquito net (1 mm mesh) were used to house groups of 20 adult female mosquitoes. In each locality, 5 houses located on the path of the vehicle were chosen to place the cages (Figure 1). Twenty minutes before ULV sprays, 5 cages were placed outside in the gardens and 5 were placed inside the houses (10 cages per locality). For each day of treatment and for each insecticide, 30 cages containing 20 Bora females were used for measuring insecticide penetration rate (n = 600 females per insecticide and per replicate). Inhabitants were asked to open their doors and windows during treatments to enable maximum penetration of the aerosol into houses. After application, cages were brought back to the laboratory for assessment of post-treatment mortality 24 hours later. During the observation period, mosquitoes were fed with sugar-soaked cotton and maintained in the laboratory at 27±2°C with a relative humidity of 80±10%.

On each occasion that spraying took place, data on the mortality of caged females of the Bora strain was complemented by data involving resistant females sampled from the population at the locality “Place d'Armes”. In each case a total of 10 cages harboring 20 females were used, with one cage on the inside and outside of the same 5 houses used to assess mortality of the Bora strain. On the 1st spray these additional cages were in the locality “Place d'Armes” and exposed to the control treatment (Banole); for the 2nd spray they were in “La Favorite” and exposed to pyrethrins (AquaPy), and for the 3rd spray they were exposed to deltamethrin (K-Othrine) in the locality “Canal”.

### Monitoring of wild *Ae. aegypti* populations

The density of adult *Ae. aegypti* population, before and after spraying, was measured by using 72 BG-Sentinel-Traps (BGS-Trap, BioGents GmbH, Regensburg, Germany) in the 9 selected localities. Traps were equipped with a mosquito attractant which was given off by the BG-Lure, a dispenser which releases a combination of lactic acid, ammonia and caproic acid, substances found in human skin [20]. Eight traps were allocated to each locality and were positioned in the same 4 houses where cage-mortality was evaluated. Four traps were placed inside houses and distributed in rooms occupied by the inhabitants, whereas the four other traps were placed outside houses on terraces where they were protected from sun and rain. Traps were deployed at 11:00 AM and collected 24 hours later. In each locality, abundance was estimated weekly starting 2 weeks before and ending 3 weeks after treatments. Abundance estimates were also made between the 1^st^ and 2^nd^ treatments.

The density of *Ae. aegypti* larvae in each locality was estimated from 7 entomological surveys. A weighted Breteau Index (WBI) was used to determine the “productivity” of female adult mosquitoes [17]. The Breteau Index is defined as the number of positive containers per 100 inspected houses. To estimate the productivity, a coefficient is attributed to each type of positive container [17], [21] to take into account the number of larvae produced in different habitats (e.g. drums [coefficient of 5.5] vs. flower pots [coefficient of 1.5]). Estimates of WBI were determined weekly, starting 2 weeks before treatments and ending 3 weeks after. Monitoring was also performed between each treatment.

### Data analysis

Mortality recorded in laboratory bioassays was corrected for control mortality by Abbott's formula [22] in case of control mortality >5%. Data were analyzed using the Analysis of Overdispersed Data (AOD) package of R which is suitable for the analysis of data based on proportions (e.g. percentage mortality) [23].

Bioassay, trap and productivity data were each analyzed using a split-plot analysis of variance (ANOVA). The bioassay and trap data showed heteroscedasticity among individual houses and so weighted means of data collected from cages either on the outside or inside of houses were calculated for each locality and day of sampling. The bioassay data was analyzed as a split-plot ANOVA with whole plots consisting of the 3 treatments blocked into 3 groups according to the entomological indices of each locality, the 1st sub-plot was defined by the 3 spray treatments, with the 2nd sub-plot depending on whether data came from cages on the inside or outside of houses. The trap and productivity data were analyzed as repeated measures split-plot ANOVA with the 6 days of sampling acting as the repeated measure. Whole plots were defined as above, with localization (inside vs. outside) forming the sub-plot for the bioassay data; these data were log (*x*+1) transformed prior to analysis, where *x* was the mean weighted value from houses within a locality. ANOVA were performed by the software JMP (v7.1) using the restricted maximum likelihood method (REML) [24].

The additional cages holding resistant females in the bioassay of cage mortality were not included in analysis above as there was no replication for each treatment on a spatial (locality) or temporal (spray) scale. Estimates for the mortality of these females are presented separately.

## Results

### Insecticide resistance status of *Ae. aegypti*

**Figure 2:**
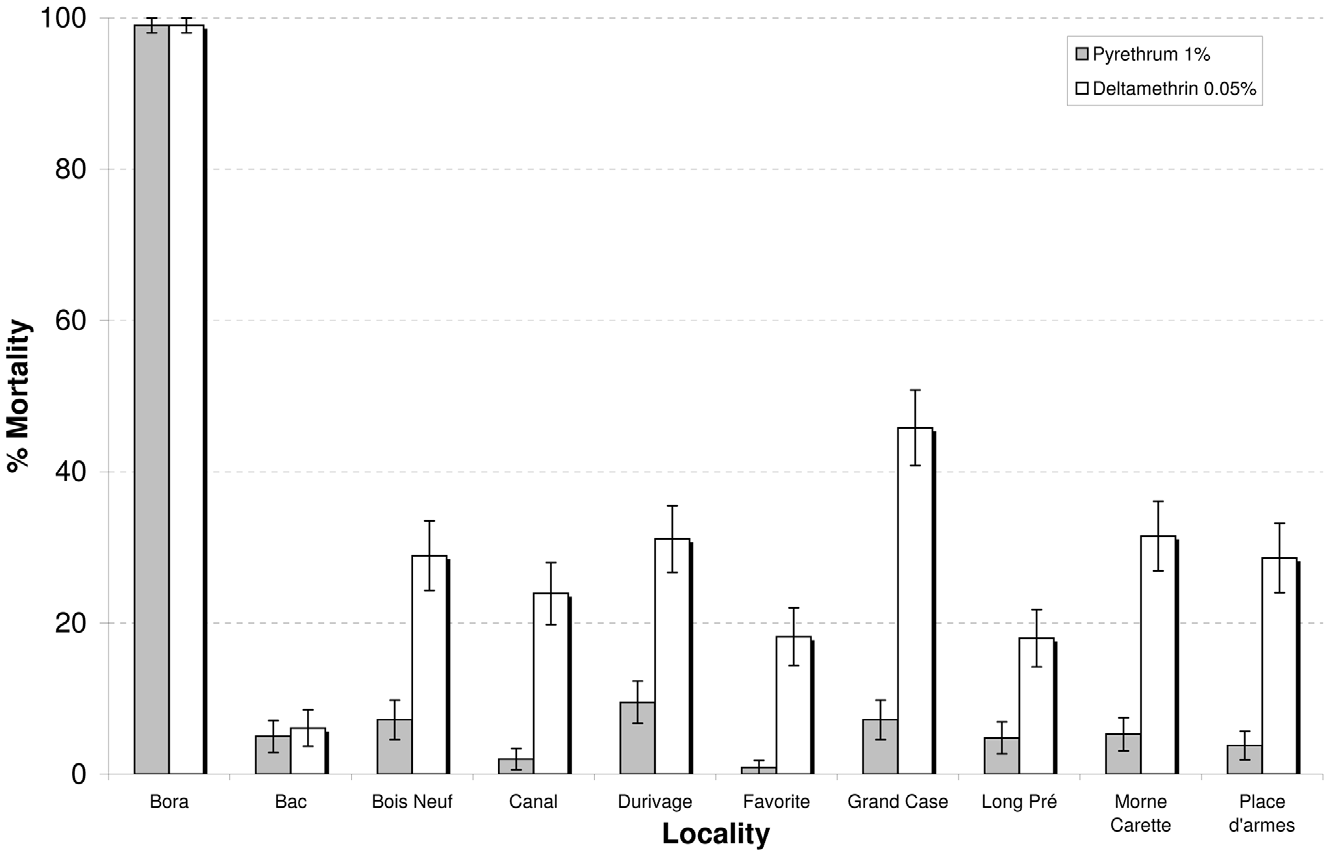
Mortality rates in WHO tube tests. This figure shows the mortality rates (± SE) of *Aedes aegypti* collected in the 9 localities selected for the space treatments and that of the laboratory susceptible strain (Bora) when exposed to pyrethrum (1%) or deltamethrin (0.05 %) in WHO tube tests. Data were analyzed using the AOD package of R.

Results obtained from WHO cylinder tests are shown in the Figure 2. The laboratory Bora strain was susceptible to pyrethrins (1%) and deltamethrin (0.5%) with 99% mortality. Mosquitoes from the 9 localities selected for the field trial showed high levels of resistance to pyrethrins (from 1 to 10% mortality) and deltamethrin (from 6 to 47% mortality).

### Efficacy of insecticide space sprays in WHO cage Bioassays

ULV sprays were performed on May 13, 18 and 20, 2009. During treatments, temperatures ranged from 25 to 31°C, relative humidity ranged from 60 to 90%, and wind speed ranged from 0 to 4 meters/second.

**Figure 3:**
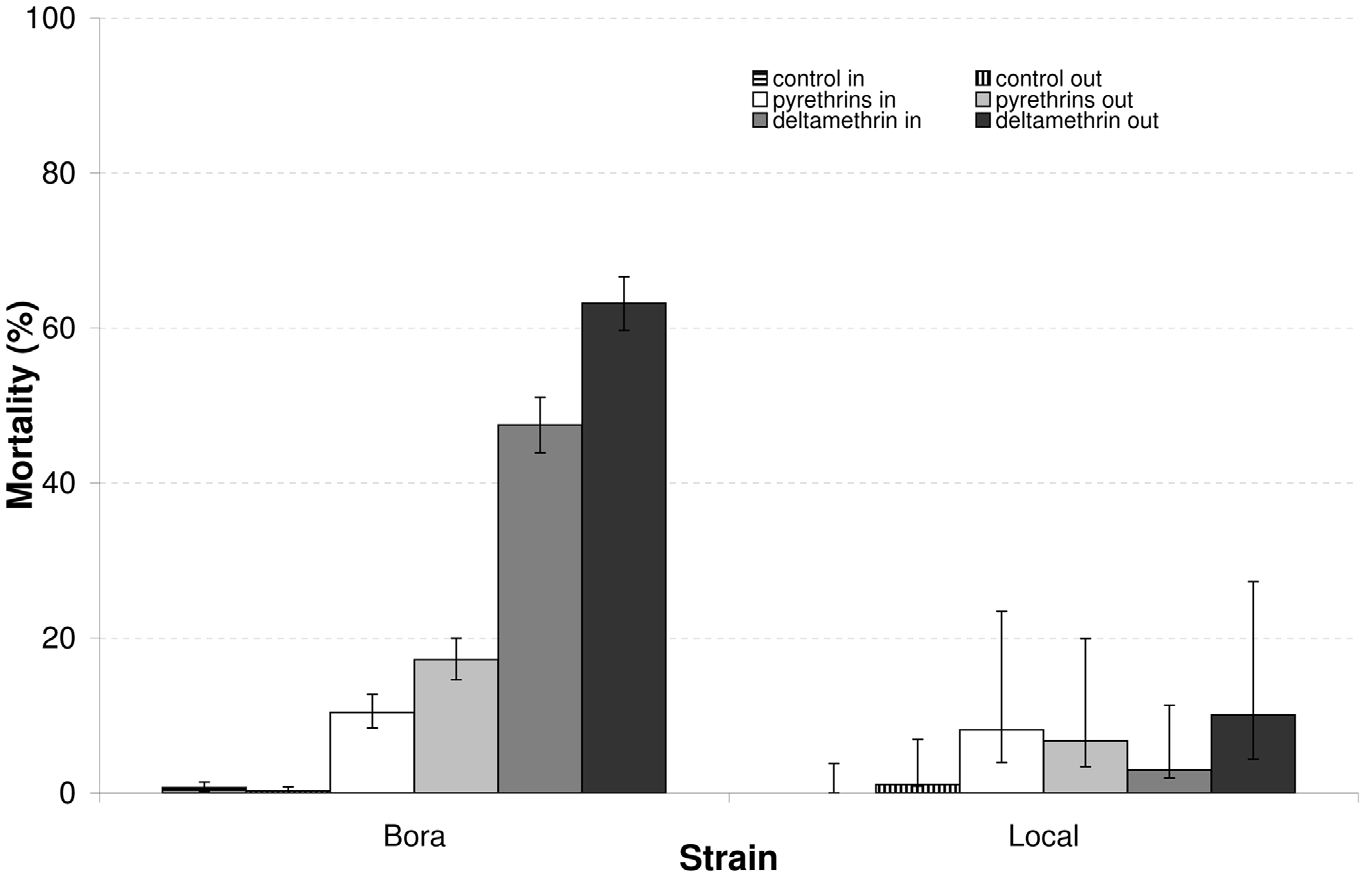
Mortality rates of wild and susceptible *Aedes aegypti* females place in sentinel cages. The first 6 columns show the estimated mortality rates from the analysis in Table 1 (± SE) of caged Bora *Aedes aegypti* females placed inside and outside the houses 24 hours post-treatment using pyrethrins, deltamethrin and mineral oil (control)*. The second 6 columns show the corresponding estimates for mortality of resistant mosquitoes (±95% CI) exposed to the same conditions.

Mortality of caged susceptible females of the Bora strain 24 hours after treatment strongly depended on the insecticide used and on whether the cages were on the outside or inside of houses (Table 1, Figure 3). Mortality in control cages was low (<1%), showing Banole W was not toxic when used alone. The mortality of females exposed to pyrethrins was 10% and 17% for those placed inside and outside the houses, respectively, and significantly lower than mortality recorded for the deltamethrin treatment. The mortality of females exposed to deltamethrin on the outside of houses was significantly higher than for those placed inside houses. These results demonstrate the efficacy of deltamethrin ULV space sprays against susceptible mosquitoes and that K-Othrine penetrates to the inside of houses. Mortality of caged resistant females did not exceed 10% for either insecticide treatment (inside or outside).

**Table 1:** Split-plot analysis of variance for the mortality of caged mosquitoes of the Bora strain exposed to different treatments*.

Source	N	DFnum	DFden	*F*	p	C.V. (%)
block (b)	3	2	-	-	-	<1.0
Treatment (T)	3	2	4	162.268	<0.001	-
b.T	9	4	-	-	-	<1.0
Spray (S)	3	2	12	3.331	0.071	-
T.S	9	4	12	0.447	0.773	-
b.T.S	27	12	-	-	-	67.2
Localization (L)	2	1	18	11.556	0.003	-
T.L	6	2	18	6.302	0.008	-
S.L	6	2	18	1.443	0.262	-
T.S.L	18	4	18	0.914	0.477	-
Residual error	54	18	-	-	-	44.6

*N  =  number, DFnum  =  numerator degrees of freedom, DFden  =  denominator degrees of freedom, C.V.  =  coefficient of variance. The data analyzed were the arcsine of the square root of mean percentage mortality per locality.

### Efficacy of space spray applications on natural *Ae. aegypti* populations

Monitoring of *Ae. aegypti* populations levels in each district were made from May through June 2009. Temperatures recorded varied from 22 to 35°C and relative humidity ranged from 55 to 98%. Daily precipitation varied from 0 to 105 mm during the evaluation with an exceptional precipitation of 300 mm on May 5^th^, 2009 one week before the 1^st^ treatment.

**Figure 4:**
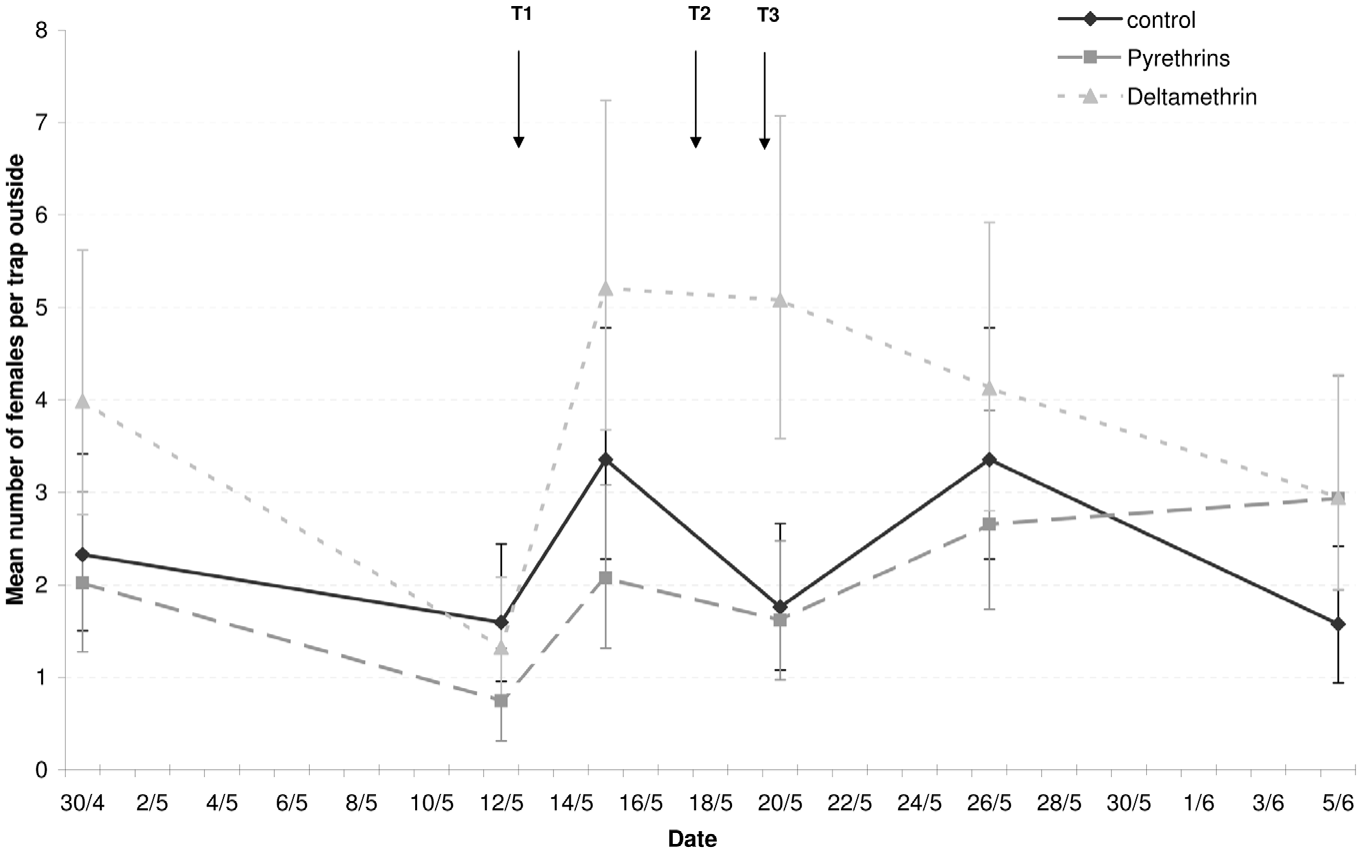
Mean number of females per trap placed outside houses before and after treatments. This figure shows estimated densities of *Ae. aegypti* females in the 9 treated sites based on trap data (see Table 2 for data analysis). Four electric traps equipped with mosquito attractant were placed outside houses on terraces where they were protected from sun and rain. Traps were deployed at 11:00 AM and collected 24 hours later. Abundance (± SE) was estimated weekly starting 2 weeks before and ending 3 weeks after treatments (T).

**Figure 5 pntd-0001202-g005:**
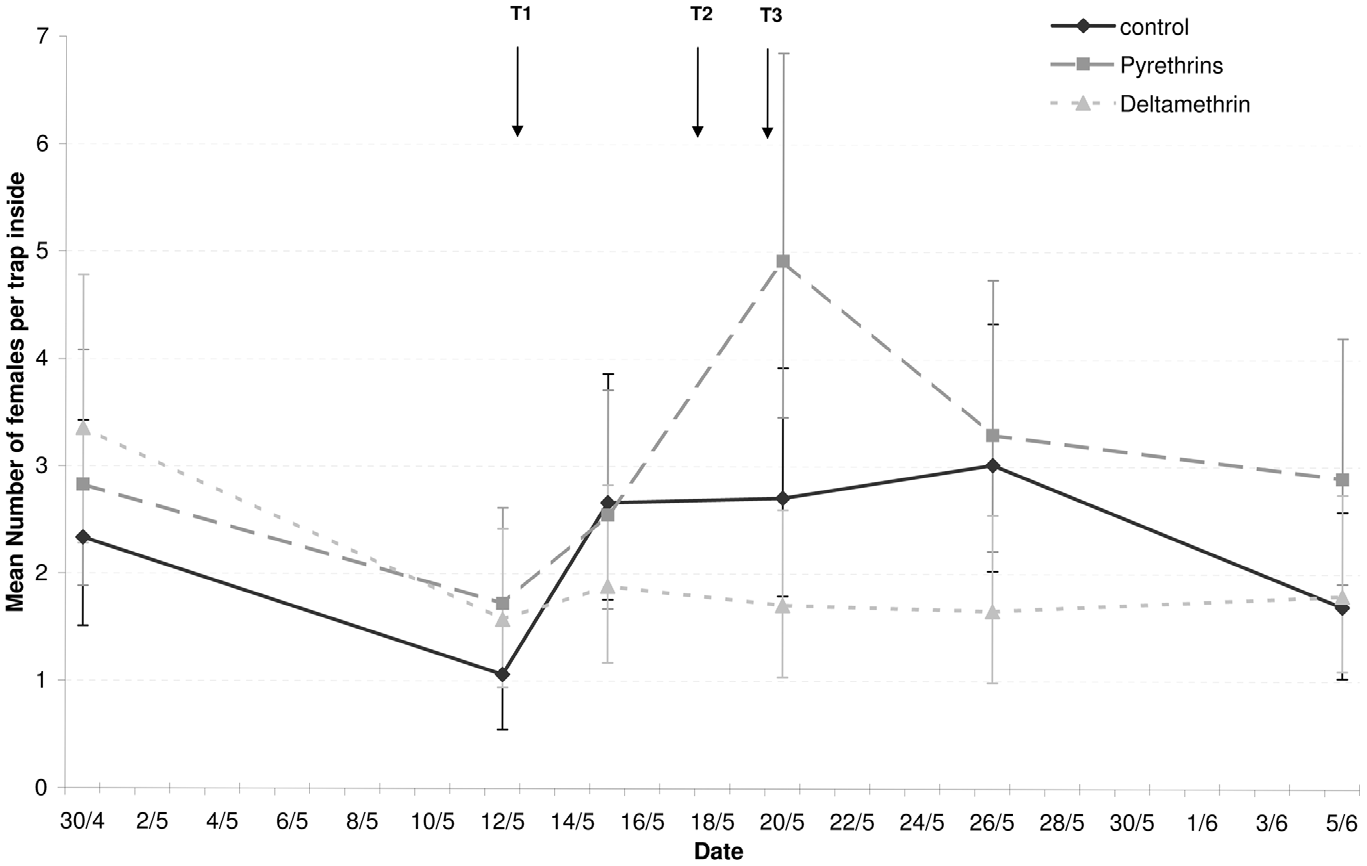
Mean number of females per trap placed inside houses before and after treatments. This figure shows the estimated densities of *Ae. aegypti* female in the 9 treated sites based on trap data (see Table 2 for data analysis). Four electric traps equipped with mosquito attractant were placed inside houses and distributed in rooms occupied by the inhabitants. Traps were deployed at 11:00 AM and collected 24 hours later. Abundance (± SE) was estimated weekly starting 2 weeks before and ending 3 weeks after treatments (T).

Estimates from the ANOVA for the numbers of females trapped on the inside or outside of houses are shown in Figures 4 and 5, respectively, grouped by day of sampling and treatment. The only significant effect found was for day of sampling (Table 2). Whereas the average number of females trapped was fairly constant over the different sampling days, it was low on the 2nd day of sampling. This day was 24 h after the application of the first spray treatments. However, it was also one week after the study area had experienced heavy rainfall. This event may have reduced recruitment into the adult population a week later due to larvae being washed out of breeding sites. Support for this argument comes from the observation that the number of females trapped on the 2nd day was also lower for locations treated with Banole (Figures 4 and 5).

**Table 2:** Repeated measures split-plot analysis of variance for the number of female mosquitoes caught in traps*.

Source	N	DFnum	DFden	*F*	p	C.V. (%)
block (b)	3	2	-	-	-	12.4
Treatment (T)	3	2	4	0.699	0.549	-
b.T	9	4	-	-	-	<1.0
Localization (L)	2	1	6	0.213	0.661	-
T.L	6	2	6	2.819	0.138	-
b.T.L	18	6	-	-	-	7,7
Day (D)	6	5	60	5.870	<0.001	-
T.D	18	10	60	0.724	0.699	-
L.D	12	5	60	0.400	0.847	-
T.L.D.	36	10	60	1.367	0.217	-
Residual error	108	60	-	-	-	82.8

*N  =  number, DFnum  =  numerator degrees of freedom, DFden  =  denominator degrees of freedom, C.V.  =  coefficient of variance. The data analyzed were the log(mean number of females +1) caught per location.

**Figure 6:**
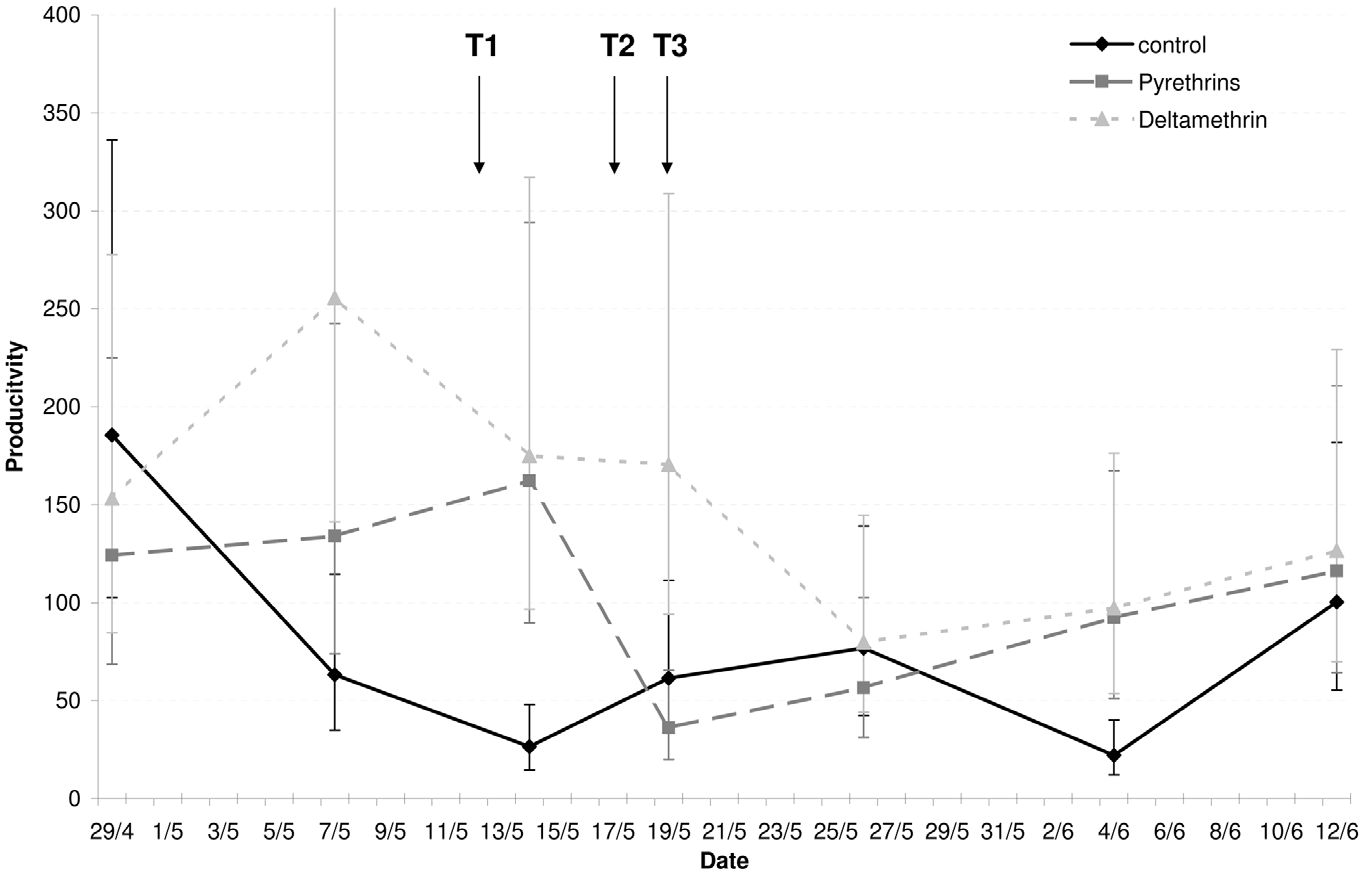
Productivity of *Aedes aegypti* in the treated areas. This figure shows the productivity (±SE) (Weighted Breteau Index) of *Aedes aegypti* of the 9 localities before and after treatments (T). The density of *Ae. aegypti* larvae in each locality was estimated from 7 entomological surveys (see data analysis, Table 3).

The application of insecticides had no detectable effect on mosquito productivity as the larval surveys found no significant differences among treatments or days of sampling (Table 3, Figure 6).

**Table 3:** Repeated measures split-plot analysis of variance for the productivity of larval sites*.

Source	N	DFnum	DFden	*F*	p	C.V. (%)
block (b)	3	2	-	-	-	31.8
Treatment (T)	3	2	4	5.063	0.0631	-
b.T	9	4	-	-	-	<1.0
Day (D)	7	6	36	1.526	0.1977	-
T.D	21	12	36	1.231	0.3006	-
Error	63	36	-	-	-	69.8

*N  =  number, DFnum  =  numerator degrees of freedom, DFden  =  denominator degrees of freedom, C.V.  =  coefficient of variance

## Discussion

This study provides the first evidence that resistance in *Aedes aegypti* can seriously reduce the efficacy of pyrethroid space sprays and their ability to control populations of the Dengue vector. Cage bioassay experiments showed that space sprays with deltamethrin at the recommended dose (i.e. 1 g/Ha) failed to eliminate natural populations of pyrethroid resistant *Ae. aegypti* (<10% mortality), whereas a relatively high proportion of susceptible mosquitoes (from 47 to 63%) were killed in the same experimental conditions. This lack of effect was confirmed by the trap data showing that 3 rounds of applying deltamethrin had no effect on the number of females mosquitoes caught in different locations (Figures 4–6). In addition, no reduction in larval indices was noted in localities treated with insecticides compared to those of the control group. Previous studies have found the penetration of insecticides to the inside of houses following aerial spraying can be limited [5], [6]. The mortality of susceptible females held in cages within houses shows the insecticide penetrated houses but these results have to be taken with caution as in natural conditions part of the mosquito population will rest in confined locations (e.g. under beds) where the insecticide may not always reach. These results suggest deltamethrin space sprays might remain effective against adult mosquitoes coming into contact with this insecticide in areas where pyrethroid-susceptible mosquitoes are present, especially if treatments are implemented during the activity period of the mosquito. However this treatment is unlikely to reduce the overall density of adult mosquitoes and virus transmission in areas where pyrethroid resistance is already present.

Synergized pyrethrins are considered as potential alternatives to synthetic pyrethroids for the control of resistant mosquitoes [19]. However the impact of this treatment against both susceptible and resistant mosquitoes was worse than for deltamethrin: it only killed relatively few caged females and caused no reduction in adult densities or larval indices after 3 rounds of application. The absence of penetration with this formulated product may be explained by an inappropriate target dose, the use of a sub-optimal spraying method (i.e. thermal fogging) and/or equipment, or a combination of these factors [7], [25]. For example, several studies have shown the efficacy of pyrethrins in reducing the incidence of West Nile virus in the USA when aerially applied (ULV) against *Culex tarsalis* and *Cx. pipiens* [26], [27]. This indicates the necessity of testing new formulations in real conditions and appropriately evaluating their performance, before regular control operations can be implemented for reducing mosquito abundance and infection rates [25].

Our laboratory bioassay showed that *Ae. aegypti* populations collected in Martinique were strongly resistant to pyrethrins and deltamethrin, confirming previous results from this area [14]. The present study showed insecticide resistance reduced the efficacy of space sprays which are the only tools available for controlling adult mosquitoes during an outbreak of dengue. Control of *Ae. aegypti* adults remains crucial to reduce the basic reproduction number (R_0_) to below a threshold were the disease cannot invade or persist in a human population [28], [29]. Early detection, with appropriate entomological indices, of localities at high risk for dengue transmission is essential for public health operators to implement early effective control especially during the extrinsic incubation of the virus [30], [31], [32]. Our study emphasizes the need to reconsider the use of pyrethroids for controlling adults in the strategy currently being implemented in Martinique. More generally, further studies should address the relationship between insecticide resistance and the efficacy of insecticide-based interventions to increase our knowledge in this poorly documented domain [8]. In addition, resistance monitoring must routinely be undertaken in all dengue control programs to help authorities and public health sector workers select the best insecticide for control of *Ae. aegypti*. Given the fact that increasing number of countries have reported alarming increases in the level of pyrethroid resistance [8], the replacement of pyrethroids by alternative molecules should be urgently addressed. The use of new chemicals with different modes of action than pyrethroids remains a feasible option. However the lack of insecticides that can be used in public health programs, due to legitimate concerns for the environment and human health, limits the options available [15]. In this context, there is an urgent need to develop strong partnerships between the public health sector and private companies to encourage investment in the research and development of new molecules, or formulations, for mosquito control [33]. However, this process is long and expensive [34].

In the short term, research on adult control should focus on the use of existing molecules. For example, the association of pyrethroid in mixtures with other insecticides with different modes of action may act synergistically and restore the efficacy of pyrethroids against resistant mosquito populations. The use of adulticides combined with larvicides (e.g. insect growth regulators with long-lasting residual activity) for spatial treatments may be a promising option during epidemics, as it can reduce simultaneously larval and adult mosquito densities [35], [36], [37], thus also making this approach cost effective [38].
